# The effect of concentrated bone marrow aspirate in operative treatment of fifth metatarsal stress fractures; a double-blind randomized controlled trial

**DOI:** 10.1186/s12891-015-0649-4

**Published:** 2015-08-20

**Authors:** Hanneke Weel, Wouter H. Mallee, C. Niek van Dijk, Leendert Blankevoort, Simon Goedegebuure, J. Carel Goslings, John G. Kennedy, Gino M. M. J. Kerkhoffs

**Affiliations:** Department of Orthopaedic Surgery, Orthopaedic Research Center Amsterdam, Academic Medical Center, Meibergdreef 9, G4-264, 1105 AZ Amsterdam, The Netherlands; Department of Surgery, Trauma Unit, Academical Medical Center, Meibergdreef 9, 1105 AZ Amsterdam, The Netherlands; The Sport Physician Group, Saint Lucas Andreas Hospital department of Sports Medicine, Jan Tooropstraat 164, 1061 AE Amsterdam, The Netherlands; Orthopaedic Surgery, Hospital for Special Surgery, 523 East 72nd Street, 5th Floor Rm 514, New York, NY 10021 USA

**Keywords:** Metatarsal, Stress fracture, Surgery, Bone marrow, Stem cells

## Abstract

**Background:**

Fifth metatarsal (MT-V) stress fractures often exhibit delayed union and are high-risk fractures for non-union. Surgical treatment, currently considered as the gold standard, does not give optimal results, with a mean time to fracture union of 12-18 weeks. In recent studies, the use of bone marrow cells has been introduced to accelerate healing of fractures with union problems. The aim of this randomized trial is to determine if operative treatment of MT-V stress fractures with use of concentrated blood and bone marrow aspirate (cB + cBMA) is more effective than surgery alone. We hypothesize that using cB + cBMA in the operative treatment of MT-V stress fractures will lead to an earlier fracture union.

**Methods/Design:**

A prospective, double-blind, randomized controlled trial (RCT) will be conducted in an academic medical center in the Netherlands. Ethics approval is received. 50 patients will be randomized to either operative treatment with cB + cBMA, harvested from the iliac crest, or operative treatment without cB + cBMA but with a sham-treatment of the iliac crest. The fracture fixation is the same in both groups, as is the post-operative care.. Follow up will be one year. The primary outcome measure is time to union in weeks on X-ray. Secondary outcome measures are time to resumption of work and sports, functional outcomes (SF-36, FAOS, FAAM), complication rate, composition of osteoprogenitors in cB + cBMA and cost-effectiveness. Furthermore, a bone biopsy is taken from every stress fracture and analysed histologically to determine the stage of the stress fracture. The difference in primary endpoint between the two groups is analysed using student’s *t*-test or equivalent.

**Discussion:**

This trial will likely provide level-I evidence on the effectiveness of cB + cBMA in the operative treatment of MT-V stress fractures.

**Trial registration:**

Netherlands Trial Register (reg.nr NTR4377)

## Background

Marines, soldiers and athletes are prone to fractures due to extensive and repeated stress on (usually weight bearing) bones [1–4]. These stress fractures develop over time and are therefore different than the more commonly accounted traumatic fractures. The damage caused by repeated forces on the bone outruns the natural remodeling process in loaded bones, resulting in a weak spot or ‘stress reaction’ and eventually a stress fracture [5–7]. Due to the increasing sports participation in the general population and awareness of their existence, these types of fractures are more frequently diagnosed. Of all stress fractures of the lower leg, the stress fracture of the fifth metatarsal has one of the highest incidences, namely up to 25 % [8]. This issue is amplified in the patients that depend on perfect physical condition like professional athletes. In a recent review of the literature, it is described that operative treatment of fifth metatarsal stress fractures results in smaller number of delayed unions or non-unions, compared to conservative treatment [9]. Still, the time to return to activity varies from 12 to 18 weeks [9–12].

Compared to a normal, traumatic fracture, stress fractures do not seem to heal via callus formation, but rather by primary bone healing with remodeling across the fracture line [7, 13, 14]. This process is slower with a higher propensity to fail, resulting in a complete fracture and refractory healing, similar to non-union [7, 14]. In traumatic fractures healing starts with formation of hematoma and therewith release of inflammatory cells. The next phase is recruitment of mesenchymal cells that differentiate into osteoblasts resulting in primary bone formation with callus production followed by secondary bone formation, with bone remodeling. In stress fractures, healing starts with the remodeling phase. Via early woven bone formation, callus is only formed along the exit point of the fracture towards the medullary cavity [15, 16].

Thus one of the major factors in bone repair seems to be an inflammatory response with the activation of Bone Marrow Stem Cells (BMSCs), which appears to be the initiating step in the cascade of bone repair. BMSCs give rise to the cells that form mesenchymal tissues like bone and cartilage [17, 18]. The exact role of BMSCs (available in cB + cBMA) in bone formation needs further clarification, but previous studies with BMSCs have provided evidence for new strategies of bone regulation [22, 24, 19], especially in delayed- and non-union fractures [20–26]. To our knowledge, there are no studies available for the role of cB + cBMA in stress fractures. In this study, the effect of adding cB + cBMA to operative treatment of MT-V stress fractures on the time to union will be assessed and compared to operative treatment alone. We hypothesize that surgery with adding cB + cBMA of MT-V stress fractures results in faster union times than surgery alone.

## Methods/Design

### Study design and informed consent

This study is a double-blind, randomized, placebo controlled, multicenter trial, which is conducted in accordance with the Declaration of Helsinki [27]. The methodology will follow the Consolidation of Standards of Reporting Trials (CONSORT) guidelines [28, 29]. Approval has been obtained from the local institutional review board Medical Ethical Commitee of the Academic Medical Center, Amsterdam (METC AMC, study reference number: NL44188.100.13 2013_182). Before study entry, all patients have to give written informed consent prior to participation.

### Randomization

Patients will be allocated to the intervention or sham-control group after signing an informed consent through a block randomization in random permutated blocks of 4, 6 or 8 patients using *“Castor edc”* [30]. Randomization will be performed online by the researcher, one day pre-operatively. At the end of the inclusion period there will be an equal number of patients in each randomization arm. Patients and X-ray assessors will be blinded to the allocation of treatment during the complete study. It is strictly forbidden for the blinded evaluator to discuss patient treatment with any study personnel. It is also strictly forbidden for study personnel to discuss treatment allocation with the patient. The key to the secured allocation data will not be broken until all patients have completed the study unless it is necessary for patient safety. This key is with an independent doctor. Patient data will be stored on hospitals’ server and only the researcher can see this data files which is stored independent of allocation data.

### Inclusion criteria

**Fig. 1:**
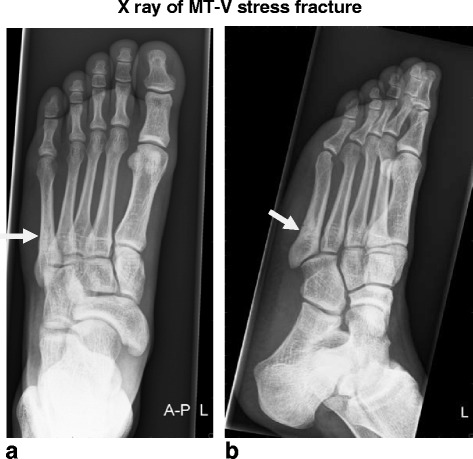
X-ray of MT-V stress fracture. **a**. AP view b. Oblique view. Stress fracture of the MT-V, located in the metaphyseal-diaphyseal junction also called zone 2; or just distally from the intrametatarsal joint (MT-IV/MT-V) in the proximal diaphysis also called zone 3 [45], with sclerosis seen on both fracture sides
- Skeletally mature

Patients will be considered to be included in this trial if they fulfill all of the following criteria:

- MT-V stress fracture diagnosed on X-ray (Fig. 1) or other radiologic imaging

### Exclusion criteria

Subjects will be excluded from this trial if they meet any of the following criteria:

- Expected non-compliance; patients who are unable to fill out questionnaires
- Cavovarus deformity of the foot as measured on the Salzmann view
- Current participation in another clinical trial
- Suffering from auto-immune disease
- Receiving biologicals, prednisolone or some kind of chemotherapy within the previous year
- Concomitant painful or disabling disease of the lower limb
- No informed consent
- Females being pregnant or nursing
- Known active malignancy
- Previous surgery for the present MT-V stress fracture

### Device description

**Fig. 2:**
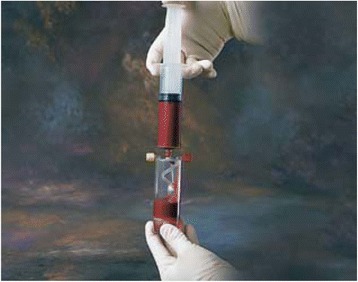
Inserting bone marrow aspirate into the device. Insert the bone marrow aspirate into the Marrow Stim device, before putting it in the centrifuge

**Fig. 3:**
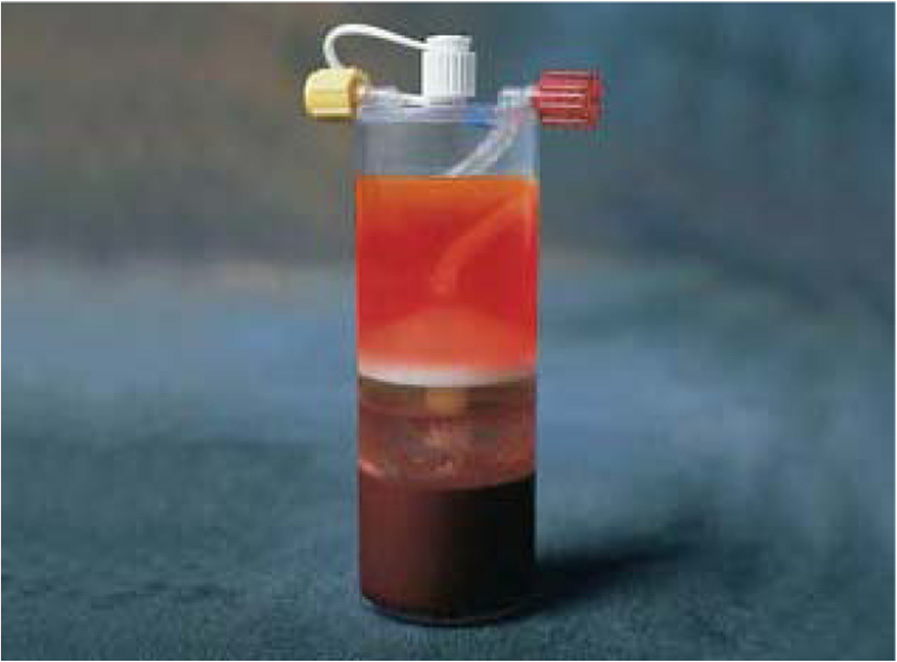
Bone Marrow Aspirate after centrifuging. Bone marrow aspirate after centrifuging (cBMA) with: *upper layer* = cell poor plasma; *middle layer* = nucleated cell concentrate; *lowest layer* = red blood cells

The bone marrow required for the cB + cBMA treatment will be harvested from patient’s own iliac crest through needle aspiration. Two 30 cc syringes will be filled with bone marrow. Utilizing the MarrowStim Concentration System® (Biomet Biologics: Fig. 2) the bone marrow aspirate will be processed in a centrifuge (together with a counterbalance, 3200 rpm for 15 min); the poor cell plasma will be separated from the nucleated cell concentrate and the cBMA is realized (Fig. 3). No bone marrow will be collected from the patients within the control group; only a sham procedure will be performed by creating a similar skin incision on the same location on the iliac crest, to maintain patients’ blinding.

### Standard treatment and investigational treatment

Experienced foot and/or ankle or trauma orthopaedic surgeons will perform the surgeries of both groups, within 2 weeks after inclusion. In brief, the proximal fifth metatarsal stress fracture will be approached and a decortication along the fracture lines will be performed. Part of this material will serve as a bone biopsy and is send for histological examination. The stress fracture will be internally fixated, with use of a cannulated intramedullary compression screw. The remaining material from the decortication will be used as an internal bone graft. The patients in the intervention group only will receive the cBMA mixed with 2 ml untreated autologous peripheral blood (cB + cBMA), together with the internal bone graft and serving as a natural scaffold; it will be put into and around the fracture. Of the 6 ml cB + cBMA obtained, 4 ml will be added to the fracture site the other 2 ml is used for analysis.

Both groups will receive a similar rehabilitation protocol for the first 8 weeks after operation; first two weeks in a non weight bearing-cast, then 2 weeks in a weight bearing cast or walker allowed to have partial weight bearing, thereafter 4 weeks in a weight bearing cast or walker without restrictions.

### Outcome measures

The primary endpoint is the time to radiographic union in weeks.

The secondary endpoints are: time to clinical union, time to return to work and/or sport (in weeks), union rate at each time point (in %), patient function and satisfaction (SF-36, FAOS, FAAM), complication rate and cost-effectiveness (SF-HLQ), compared between intervention and control group.

During the operation a bone biopsy is taken. This will be histologically analyzed. From the obtained cBMA a 2 ml sample will be analyzed (flow cytometry and a multiplex assay for detecting CD 31, CD 34, CD 45, CD 90, CD 105, CD 166 amounts, CFU-F assay and mRNA analysis).

### Objectives and hypothesis

The main objective is to determine the effect of cB + cBMA on healing of surgically treated fifth metatarsal stress fractures. We hypothesize that intramedullairy screw fixation with cB + cBMA will generate a shorter time to union. Furthermore we hypothesize that the total costs are lower and that there will be a quicker return to sports or activities.

## Definitions

### Primary outcome

The primary outcome will be assessed on X-ray in different directions; on AP view, lateral view and an oblique view. The following will be scored by two single blinded evaluators (skeletal radiologist and orthopaedic surgeon): correct position osteosynthesis (yes/ no), fracture line visible (yes/ no), % of fracture line visible, union (yes/ no).

The X-rays will be acquired every 2 weeks until the 14^th^ week and, after that, only in the patients in which radiological union has not yet occurred as deemed necessary by the treating physician.

### Secondary outcomes

Clinical union will be measured during physical examination. Pain on axial compression force and pain on the fracture site during palpation will be monitored through a patient-based VAS score. When patients still report significant pain, measured as a VAS > 3 on a Visual Analogue Scale from 0 to 10 [31, 32], clinical union is not yet achieved. Return to sports is defined as the time (in weeks) to resumption of weight-bearing sports after surgery and the level should be maintained for at least 30 days [33]; return to work is defined in the same way. When returned to sport, but activity level decreases to below the start level within 30 days after resumption, then this will be deemed as a failed return to activity and the date will not be counted. From the 6^th^ till the 14^th^ week, most unions are expected to occur. On each of these biweekly time points a union rate will be counted; number of unions at t = XXX divided by total sample size (%).

The SF-36 is a patient-administered, generic health related quality of life instrument [34, 35]. The FAOS was developed to assess the patients’ opinion about a variety of foot and ankle-related problems [36]. The results can be presented as an outcome profile. The FAAM is a 29-item questionnaire. The FAAM consists of 2 subscales: Activities of Daily Living and Sports [37].

Cost-effectiveness is analyzed using the The EuroQol (EQ-5D) [38, 39] and the SF-36, FAOS, FAAM as measurement for effectiveness of treatment. Costs are calculated from the SF HLQ [40, 41] and the cost of the investigated intervention. The questionnaire consists of 11 questions and provides information about labor (missing) costs and additional paid working hours.

## Results

### Adverse events

Any (serious) adverse event during the trial period will be recorded. Adverse events are defined as any undesirable experience occurring to a patient receiving an investigational medical device that does not necessarily have to have a causal relationship with the device under investigation, e.g. infection, numbness, or paraesthesia. A serious adverse event (SAE) is any undesirable experience associated with the use of the investigational treatment that results in death, is life threatening (at the time of the event), requires hospitalization or prolongation of existing inpatients’ hospitalization, or results in persistent or clinically relevant disability or incapacity. All SAEs will be reported to the local Medical Ethics Committee within 15 days according to the regulations.

### Data collection

Data for this clinical trial will be collected and documented on specially designed subject Case Report Forms (CRFs) provided digitally. Authorized study site personnel will complete CRFs only. CRFs will be reviewed and signed by the Investigator or his/her designees. Since there is a potential for errors, inaccuracies, and misinterpretation in transcribing data onto the CRFs, the following documents are available at all times for inspection and comparison to the CRFs by the study monitor where appropriate: data query forms, originals and certified copies of all relevant records and reports, copies of test results. On CRFs and patients’ cB + cBMA samples, patients will not be identified by their names but by an anonymized code. The subject identification code list will be safeguarded by the investigator only.

### Data acquisition and follow-up

Participating patients will be assessed at the following time points (Table 1):

**Table 1:** Schedule for screening and follow-up of included participants

Screening and Follow-Up Clinical and Radiographic Exams						
Action	Pre-operative	Intra-operative	Follow-Up Visit (biweekly 2-14 weeks)	Follow-Up Visit Until Union (biweekly) (>14 weeks, determined by physician)	6 month follow-up visit	1 year Follow-Up visit
Information letter	X					
Obtain written informed consent	X					
Complete eligibility checklist	X					
Complete medical history and baseline characteristics (form)	X					
Complete physical examination (form)	X		X	X	X	X
Surgery		X				
- Operative form (surgical information)		X				
- cB + cBMA preparation and administration		X				
- Bone biopsy fracture site		X				
Lab assessments	X					
Questionnaires	X		X		X	X
Radiographic assessment	X	X	X	X	X	X
Complication (form)	X	X	X	X	X	X
Adverse events, withdrawals & re-operation (form)	X	X	X	X	X	X

- before inclusion: information letter, complete reliability checklist
- preoperatively (after an enrollment period of two weeks): informed consent, complete reliability checklist, baseline characteristics (age, gender, weight, height, affected side, duration of symptoms, smoking status, AAS before symptoms and at preoperative assessment, past medical history, medication used), type of sport and profession, local physical examination with VAS, laboratory assessment (Hemoglobin levels, leucocytes, sodium, potassium, urea and creatinine), FAOS, FAAM, SF-36, EQ-5D, SF HLQ, X-ray (AP oblique and lateral);
- intraoperative: cB + cBMA preparation and administration, bone biopsy of the fracture site, right after internal fixation an X ray (AP, oblique and lateral) and an operative form (surgical information) with complications and (S)AEs if applicable;
- biweekly 2-14 weeks postoperatively: wound inspection, local physical examination with VAS, FAOS, FAAM, SF-36, EQ-5D, TiC, X-ray (best view for analyzing the fracture), resumption of sports and work, complications and (S)AEs;
- six months postoperatively: local physical examination with VAS, FAOS, FAAM, SF-36, EQ-5D, TiC, X-ray (best view for analyzing the fracture), resumption of sports and work, complications and (S)AEs;
- one year postoperatively: local physical examination with VAS, FAOS, FAAM, SF-36. EQ-5D, TiC, X-ray (best view for analyzing the fracture), resumption of sports and work, complications and (S)AEs

If union is not yet achieved after 14 weeks, patients are seen as determined by the responsible physician. At every subsequent visit at least an X-ray is made to be able to evaluate the status of the fracture (primary outcome).

## Withdrawal of subjects

Subjects can leave the study at any time for any reason if they wish to do so, without any consequences. The investigator can decide to withdraw a subject from the study for urgent medical reasons. Subjects will not be replaced after withdrawal. Data of a withdrawn subject will be used, but we will delineate from which visit (time point) data is incomplete and (if known) why the subject is sequestered. To investigate why subjects are withdrawn, they will be contacted by telephone and/or letter to assure the reason for their withdrawal and to ask them to come in for an optional final visit.

## Sample size

The sample size calculation was performed based on time to radiological union. Significance level was set at p < 0,05. In recent studies, the mean time to union was 12 weeks [11, 12]. We argue that reducing this with 2 weeks would be clinically relevant: Group 1 (without cB + cBMA) has a mean of 12 weeks, group 2 (with cB + cBMA) a mean of 10 weeks. As the standard deviation is difficult to determine, we estimated SD with use of the method described by Walter and Yao [42], which resulted in an SD of 1.88 weeks.

To achieve a power of 90 % (with an effect size of 1,06) we need a number of 20 patients in each group. Taking into account a possible dropout-rate of maximally 20 %, we need 25 patients in each randomization arm. This calculation will be validated and altered as needed after the interim analysis according to O’Brien [43] and performed when 10 patients enrolled in each arm (40 %) have reached the 14 weeks’ time point. This analysis reduces the significance of the final comparison of the primary outcome to *p* = 0.049. When a difference of ≥3 weeks between the means of the primary outcome among the groups is detected (SD 1.88) at the interim analysis; a significance is already found (*p* = 0.003).

## Statistical methods

An online, quality-assured, digital database will be created containing the data. The analyses will be performed with SPSS statistical package (version 17.0; SPSS, Chicago, Illinois). Patient’s demographics, gender, age, height, weight, affected side will be described for both treatment groups and safety will be assessed by identifying and summarizing complications and other clinically relevant adverse events collected throughout the study. Continuous data will be presented as the mean (and standard deviation) if normally distributed (according to Fischers exact or eyeball test); otherwise, the medians (with range) will be reported. Dichotomous data will be presented as frequencies (with accompanying percentages).

To check the distribution of the baseline characteristics, the treatment groups will be compared using Student’s t-tests or chi-square tests, depending of the type of outcome measure.

The primary endpoint – number of weeks until patients achieved radiological union postoperatively – will be analyzed using a student’s *t*-test or equivalent; this parameter will be used for clinical union as well. A chi-square test will be used to analyze union rate at each time point, and a student’s *t*-test will be used to determine statistical differences in time to return to work and/or sport. Patient-reported outcome measures will be analyzed using a student’s *t*-test or repeated measures ANOVA.

Risk factors for the development of healing problems (such as smoking behavior, BMI, and age) will first be identified using univariate analysis (t-tests, chi-squared tests). In case of significance (significance level will be set at 0.1), the risk factors will be entered in a multivariate logistic regression model. The cBMA analysis will be reported as absolute cell count and in % of cell types. Data on the bone biopsies of the stress fractures will be descriptive.

A comprehensive, updated sample size justification and statistical plan will be created together with an interim report after the interim analysis.

## Quality assurance

A clinical research associate from our Clinical Research Unit will monitor the trial. Monitoring will consist of 100 % check informed consent procedure, registration of adverse events, completeness of the trial master file, and verification of source data (primary outcome in a 10 % sample).

## Public disclosure and publication policy

This trial has been registered in the Netherlands Trial Register (NTR4377), and about to start including patients and data collection. Publication will be in accordance with the basic principles of the International Committee of Medical Journal Editors on publication policy [44]. The writing committee will consist of the following people: H. Weel, L. Blankevoort and G.M.M.J. Kerkhoffs. All other individuals who made and will make substantial scientific contributions to the conduction of the trial and to the final manuscript will be listed as an author. Individuals who make substantial contributions to the conduction of the trial will be acknowledged at the discretion of the writing committee.

## Discussion

In this paper the rationale for the study and the protocol for conducting a double-blind, randomized, controlled trial on the effectiveness of adding cB + cBMA to the operative treatment of MT-V stress fractures are described.

Time to union is the primary outcome measure. This is not easy to be objectively determined, therefore 2 experienced, blinded observers (experienced skeletal radiologist and orthopaedic surgeon) will assess this outcome.

This trial will not only contribute to the knowledge of effectiveness of bone marrow cells in stress fractures. Because the consistence and consistency of the cBMA product is also measured, we will learn more about which working mechanisms and osteoprogenitor support of the bone marrow cells are presented in fracture healing. Given the relatively minimally invasive intervention of aspirating autologous bone marrow, its simple and fast concentration into cBMA and the easy way of adding it locally, it has high potential to provide a safe and effective additional treatment option for stress fractures and other fractures predisposed for delayed- or non-union.

Additionally, the results of this study could also help in a better understanding of the healing pathways of stress fractures in general. Theretofore, a biopsy is taken from all fractures to be histologically analysed, because the healing pathways in stress fractures are still underexposed.

## Abbreviations

SF-36: Short Form- 36
FAOS: Foot Ankle Outcome Score
FAAM: Foot Ankle Activity Measurement
SF HLQ: Short Form Health Labor Questionnaire
MT-V: Fifth Metatarsal
cB + cBMA: concentrated Blood and concentrated Bone Marrow Aspirate
AE: Adverse Event
SAE: Serious Adverse Events
